# Neonatal ECMO Study of Temperature (NEST) - a randomised controlled trial

**DOI:** 10.1186/1471-2431-10-24

**Published:** 2010-04-19

**Authors:** David J Field, Richard Firmin, Denis V Azzopardi, Frances Cowan, Edmund Juszczak, Peter Brocklehurst, the NEST Study Group

**Affiliations:** 1 Department of Heath Science, Department of Health Sciences, University of Leicester, 22-28 Princess Road West, Leicester, UK; 2 ECMO Department, Glenfield Hospital, Groby Road, Leicester, UK; 3 Division of Clinical Sciences, Faculty of Medicine, Imperial College London, Hammersmith Campus, Du Cane Road, London, UK; 4 Hammersmith Hospital, Du Cane Road, London, UK; 5 National Perinatal Epidemiology Unit, University of Oxford, Old Road Campus, Oxford, UK

## Abstract

**Background:**

Existing evidence indicates that once mature neonates with severe cardio-respiratory failure become eligible for Extra Corporeal Membrane Oxygenation (ECMO) their chances of intact survival are doubled if they actually receive ECMO. However, significant numbers survive with disability. NEST is a multi-centre randomised controlled trial designed to test whether, in neonates requiring ECMO, cooling to 34°C for the first 48 to 72 hours of their ECMO course leads to improved later health status. Infants allocated to the control group will receive ECMO at 37°C throughout their course, which is currently standard practice around the world. Health status of both groups will be assessed formally at 2 years corrected age.

**Methods/Design:**

All infants recruited to the study will be cared for in one of the four United Kingdom (UK) ECMO centres. Babies who are thought to be eligible will be assessed by the treating clinician who will confirm eligibility, ensure that consent has been obtained and then randomise the baby using a web based system, based at the National Perinatal Epidemiology Unit (NPEU) Clinical Trials Unit. Trial registration.

Babies allocated ECMO without cooling will receive ECMO at 37°C ± 0.2°C. Babies allocated ECMO with cooling will be managed at 34°C ± 0.2°C for up to 72 hours from the start of their ECMO run. The minimum duration of cooling will be 48 hours. Rewarming (to 37°C) will occur at a rate of no more than 0.5°C per hour. All other aspects of ECMO management will be identical. Primary outcome: Cognitive score from the Bayley Scales of Infant and Toddler Development, 3^rd ^edition (Bayley-III) at age of 2 years (24 - 27 months).

**Discussion:**

For the primary analysis, children will be analysed in the groups to which they are assigned, comparing the outcome of all babies allocated to "ECMO with cooling" with all those allocated to "ECMO" alone, regardless of deviation from the protocol or treatment received. For the primary outcome the analysis will compare the mean scores for each group of surviving babies. The rationale for this choice of primary analysis is to give a fair representation of the average ability of assessable children, accepting the limitation that excluding deaths might impose.

The consistency of the effect of cooling on the group of babies recruited to the trial will be explored to see whether cooling is of particular help, or not, to specific subgroups of infants, using the statistical test of interaction. Therefore pre-specified subgroup analyses include: (i) whether the ECMO is veno-arterial or veno-venous; (ii) whether the child's oxygenation index at the time of recruitment is <60 or ≥ 60; (iii) initial aEEG pattern shown on the cerebral function monitor, and (iv) primary diagnostic group.

**Trial Registration:**

Current Controlled Trials ISRCTN72635512.

## Background

### Introduction

NEST is a multi-centre randomised controlled trial designed to test whether, in neonates requiring ECMO, cooling to 34°C for the first 48 to 72 hours of their ECMO course leads to improved later health status. Infants allocated to the control group will receive ECMO at 37°C throughout their course, which is currently standard practice around the world. Health status of both groups will be assessed formally at 2 years corrected age.

### Hypothesis

Cooling neonates requiring ECMO to 34°C for the first 48 to 72 hours of their ECMO run results in improved neurodevelopmental outcome at 2 years corrected age. (Note: neonate is defined as less than or equal to 28 days of age).

### Background

ECMO is an invasive method of life support used in severe respiratory or cardio-respiratory failure. ECMO entered clinical practice as an extension of the technology that produced cardiopulmonary bypass. Although it has been used in patients of all ages, relatively mature (35 weeks gestation age or more) newborn infants form by far the largest patient group to date.

In the last 20 years ECMO has been used to support over ten thousand neonates with severe cardio-respiratory failure worldwide. Despite the availability, in recent years, of new therapies the majority of neonates who develop the severest forms of cardio-respiratory failure still require ECMO. The most recent evidence indicates that ECMO is the most effective form of life support for neonates that fulfil the eligibility criteria for ECMO both in terms of improved survival and morbidity [1]. However, outcome data from both randomised trials and clinical series indicate that ECMO eligible neonates are at high risk of later neurodevelopmental problems. Approximately 40% of survivors develop some form of impairment irrespective of whether they received ECMO or some other less invasive means of life support [2]. Data from the UK collaborative ECMO trial indicates that 32% of the original cohort of children in the ECMO arm survived without disability at 4 years of age compared with 14% in the conventional treatment arm [2]. Although two thirds of the children seen at 4 years were described as having a cognitive outcome within the normal range, the mean general conceptual ability score was only 93, significantly below average. Specific difficulties in visiospatial tasks as well as behavioural problems and sensory losses were also found.

#### Causation of brain injury in infants who meet the criteria for ECMO

For most infants the precise aetiology of the neurological damage associated with severe cardio-respiratory failure is uncertain. Infants who meet the entry criteria for ECMO are, by definition, relatively mature and do not have evidence of severe neurological compromise at birth. However, there is often a history of fetal distress, meconium stained liquor and infection. Sub-optimal Apgar scores and low cord pH values are common [3]. Postnatally, they may be acidotic, poorly perfused and exposed to varying degrees of hypoxia. Severe cardio-respiratory disease may be associated with a persistent fetal circulation and a patent foramen ovale, leaving the cerebral circulation vulnerable to circulating emboli and thrombus [4]. However, the time of maximal physiological derangement from the underlying condition leading to the severe cardio-respiratory failure normally coincides with a decision to use ECMO and the establishment of ECMO poses a further insult to the cerebral circulation and hence to the brain [5]. Thus the establishment of ECMO may act as a marker for a time of "maximum risk" for the brain and it would appear appropriate to attempt to protect the brain at this critical period.

#### The pattern of brain injury and likely outcome in infants meeting the criteria for ECMO

The existing data [6-9] on brain imaging following ECMO confirm the above i.e. that the predominant pattern of injury is white matter infarction with cortical involvement. Haemorrhage occurs to a lesser extent. These observations are consistent with the fact that these infants do not have very low Apgar scores and severe acidosis and they are not thought to be encephalopathic. Therefore it is unlikely that injury to the central grey matter will be the predominant pattern of injury. Given the background problems these infants are exposed to, their neurological deficits are likely to result from a more prolonged sub-acute insult leading to white matter and cortical damage in a parasagittal distribution. Data from studies in term infants with neonatal encephalopathy or seizures with this pattern of damage suggest that unless the damage is very severe, the children do not develop cerebral palsy. However they do have delays in acquisition of motor skills, difficulty with fine motor tasks, visual and visuospatial difficulties, some delay in the development of speech and language, learning and behavioural difficulties and sub-optimal head growth [10-12]. Children with focal infarction have a motor outcome consistent with the site of injury [13,14]. The existing data on outcome in children meeting ECMO criteria are consistent with this description [2].

#### Rationale for the use of mild hypothermia

One of the most promising methods for achieving neuroprotection is the use of mild hypothermia. This approach has been investigated in animal studies by a number of groups using a variety of "hypoxic-ischaemic" insults [15-19]. These studies, including those by members of our own group, have consistently shown benefit without adverse side effects, even when hypothermia was applied up to 6 hours after the injury. Reduction of body temperature by 3-4°C after a cerebral insult was associated with improved histological and long-term behavioural outcome in both adult and newborn animals. Preliminary clinical studies in adult humans of whole body hypothermia following head injury, cardiac arrest and stroke suggested potential benefit in all these groups. However, results of randomised trials in adults have shown beneficial effects in relation to cardiac arrest but not head injury [20]. Studies in human neonates who have been subject to hypoxic-ischaemic insults suggest that the technique is safe [21-23]. Preliminary results from the first two randomised trials to investigate the potential for mild hypothermia applied for 72 hours from within six hours of birth to protect the neonatal brain after a perinatal hypoxic-ischaemic insult have suggested some evidence of benefit [24,25]. These data suggest that neonates with the most severe cerebral damage may be less likely to benefit from cooling. Neonates with this type of damage are excluded from ECMO. However, outcomes for those with the less severe insults as assessed by pre-hypothermia aEEG recordings had evidence of an improved outcome [26]. Further randomised trials to assess the effects of hypothermia on the long term outcome of such infants are still on-going. It is likely that a number of such trials will be needed to fully evaluate the potential of hypothermia in reducing brain injury, as there is evidence that the effectiveness of the intervention, if present, is affected by a variety of factors such as the timing of onset of hypothermia in relation to the insult and the degree and duration of the hypothermia.

The focus of most animal and clinical studies into brain damage in the relatively mature newborn has been in relation to a hypoxic-ischaemic insult around the time of birth leading to hypoxic-ischaemic encephalopathy. Following such an insult brain cells are lost in two phases: neuronal loss may occur during or immediately following the insult and subsequently further cells die over weeks or months primarily as a result of apoptosis. It is this second phase of cell loss that early mild hypothermia appears to have the potential to influence [27]. There are no equivalent studies to confirm that apoptosis is an important component of the brain damage and poor outcome noted in ECMO survivors. However, given the nature of the illnesses these children suffer in order to become ECMO eligible and the type and degree of physiological derangement, there is clearly potential for both types of neuronal loss described above to occur. There is clear evidence that infants who reach ECMO eligibility criteria have a high rate of neurodisability if they survive. The risk for neurodevelopmental impairment is significantly reduced if the newborn actually goes on to ECMO but the rate of adverse outcome remains high. Whilst the nature and timing of the brain insult(s) that lead to this adverse outcome remain poorly understood, given the background on which they occur, it is appropriate to formally test the potential of hypothermia to reduce this risk.

#### Feasibility studies for hypothermia in infants having ECMO

Although there is some information about the physiological impact of mild hypothermia [33°C or above) data applicable to the newborn are very limited. More information is available in relation to moderate hypothermia in adults but this is only helpful in identifying those bodily functions that might be impaired by a period of mild hypothermia [28-30]. No systematic studies have previously been performed to assess the therapeutic affect of mild hypothermia on patients on ECMO. Therefore in preparatory work (funded by The British Heart Foundation) we have investigated the feasibility and safety of using mild hypothermia in neonates receiving ECMO. The study population of this investigation compromised the following infants (note the next reduction in temperature or increase in duration of hypothermia only occurred after the successful completion of the previous stage):(Neonatal patients were defined as infants of less than 28 days of age).

5 neonates maintained at 37°C throughout their ECMO course (controls);

5 neonates maintained at 36°C for the first 12 hours of their ECMO course;

5 neonates maintained at 35°C for the first 12 hours of their ECMO course;

5 neonates maintained at 34°C for the first 12 hours of their ECMO course.

5 neonates maintained at 37°C throughout their ECMO course (controls);

5 neonates maintained at 36°C for the first 24 hours of their ECMO course;

5 neonates maintained at 35°C for the first 24 hours of their ECMO course;

5 neonates maintained at 34°C for the first 24 hours of their ECMO course.

5 neonates maintained at 34°C for the first 48 hours of their ECMO course.

The decision to use ECMO was based on standard criteria and was made on clinical grounds alone (an oxygenation index of 40 or more, or a PaCO_2_of 13 kPa or more despite maximal non-ECMO support and with no contra-indication to ECMO).

The clinical course of each infant was closely scrutinised but in addition a number of specific investigations and observations were performed in relation to: blood clotting, immune function, EEG, cardiac conduction, oxygenator function and circuit performance.

A paper describing the first 20 patients [31] and a second paper focusing on the next 25 patients have been published [3]. In summary, there was no evidence that hypothermia had an adverse impact on the acute clinical condition of the baby. There did appear to be an effect on cardiac conduction as measured by ECG, but this was not clinically apparent during the baby's ECMO course. There was no suggestion of an excess of haemorrhagic complications. Similarly there was no adverse effect noted in relation to the ECMO circuit or its management. Of particular relevance to long term outcome, temperature change had no effect on the aEEG.

## Methods/Design

This is a pragmatic multi-centre randomised controlled trial. Babies will be randomised to one of two arms:

The treatment arm in which babies will be cooled to 34°C for the first 48 to 72 hours of the ECMO run.

The control arm in which babies will undergo conventional (normothermic) ECMO.

### Inclusion criteria

Babies recruited to the study must meet the existing standard criteria for ECMO eligibility. These include:

• at least 35 weeks gestation;

• at least 2000 g weight;

• no uncontrolled bleeding disorder;

• no congenital or acquired CNS disorder;

• no more than 7 consecutive days of high pressure ventilation prior to referral for ECMO;

• the underlying condition is potentially reversible;

• evidence of severe cardio-respiratory failure;

• less than 29 days of age.

### Exclusion criteria

• All neonates referred with diaphragmatic hernia;

• All neonates receiving ECMO for post operative cardiac support.

• All neonates who have been cooled prior to ECMO.

Among the babies with diaphragmatic hernia referred for ECMO a number have severe pulmonary hypoplasia incompatible with survival. These infants cannot be reliably detected prior to ECMO and there is no rationale for believing that cooling will help these infants [32]. Babies receiving ECMO following cardiac surgery, in general, are not comparable in terms of their risk of serious adverse neurodevelopmental outcome.

All other infants will be eligible unless aspects of their medical condition prevent or render them inappropriate for ECMO.

### Duration of cooling

At the time of the pilot studies underpinning this trial, two factors were considered of particular importance when deciding on the duration of cooling to be used as the intervention:

1. Virtually all babies requiring ECMO have a run of at least 48 hours (i.e. cooling for 48 hours could be used without altering other aspects of normal ECMO management).

2. Previous studies had found that cooling for 48 hours could have a therapeutic effect.

However, subsequently two randomised trials of mild cooling in infants with hypoxic-ischaemic encephalopathy found cooling for 72 hours may result in improvement in outcome [25,26]. Cooling for this duration has no effect on the ECMO circuit as patients are sometimes maintained at this temperature for prolonged periods for reasons other than neuroprotection e.g. reducing oxygen consumption. Although the majority of babies on ECMO require at least 72 hours of ECMO, some babies can come off ECMO at between 48 and 72 hours. To come off ECMO while maintaining cooling would be technically complex and might result in adverse consequences. However, to maintain babies on ECMO longer than they would normally require in order to maintain cooling for 72 hours would mean exposing the baby to the on-going risks of ECMO unnecessarily. Therefore, the duration of the intervention in NEST will be for a minimum of 48 hours and a maximum of 72 hours. Infants will be re-warmed before coming off ECMO.

### Consent

Neonates will be recruited only after informed consent has been obtained from the parent(s). Clearly this will pose major logistic problems since it is planned that cooling will commence at the start of the ECMO run and in many cases there will be an urgent need to establish ECMO. In order to optimise recruitment:

• ECMO centres involved in the study will discuss the study with the parents of all children cared for "in house" who are likely to need ECMO e.g. children with persistent pulmonary hypertension and deteriorating blood gases despite full conventional support. These discussions will be supported by information leaflets for parents describing both ECMO and the study.

• Where neonates are "retrieved" for ECMO from other centres the situation will be more difficult. In many cases there will be a period (of up to 2 days) during which a transfer for ECMO is being considered. In these circumstances the ECMO centre will have the opportunity to fax ahead detailed information about the study and also brief the local clinical team prior to any retrieval. Should the referral for ECMO subsequently go ahead, and parents agree to their child joining the study, formal consent can be obtained by the routine retrieval team without undue haste. On other occasions such referrals and transfers for ECMO take place at short notice. On these occasions the team will include sufficient personnel (an ECMO specialist, or doctor or senior nurse involved with the ECMO programme) to allow time to be spent with the parents both to explain the study and obtain informed consent. At the start of the study, ECMO centres will also discuss with clinicians at centres that have previously referred babies for ECMO, the details of the study protocol.

The actual consent procedures will involve a two stage process. At the time of initial recruitment parents will receive the Information Leaflet about the study (as well as the leaflet explaining ECMO produced by the ECMO centre retrieving or caring for the baby) and at that time parents will be asked to give written consent. Where it is not possible for parents to travel with the baby to the ECMO centre, consent may be obtained over the telephone. This will be witnessed by a second member of staff and the Telephone Consent form completed. Both members of staff will sign the Telephone Consent form and on arrival at the ECMO centre parents will be asked to add their signature to confirm their consent. Where parents do not have a good grasp of English, unless a good interpreter is immediately available, recruitment should not proceed. Where a child is recruited, a follow up conversation (using the Further Information Leaflet about the study) should take place during the stay in the ECMO centre. No "re consenting" is required but the conversation and the parent's agreement to allow their baby to continue in the study should be documented in the medical records.

### Randomisation

All infants recruited to the study will be cared for in one of the four UK ECMO centres. Infants who require ECMO will be transferred to these centres from across the country. Babies who are thought to be eligible will be assessed by the treating clinician who will confirm eligibility, ensure that consent has been obtained and then randomise the patient using the web based system, provided by the National Perinatal Epidemiology Unit (NPEU). As soon as parental consent has been obtained, the recruiting ECMO specialist, or delegate, will log on to the web site and obtain the treatment allocation, which will be either to "ECMO" or "ECMO with cooling". Minimisation will be used to ensure balance between the groups with respect to the most important factors likely to affect later outcome, one of which is the method of cannulation (either veno-venous or veno-arterial).

### ECMO management

In those babies randomised to "ECMO with cooling", cooling will begin immediately upon initiation of the extra-corporeal circulation. The baby's temperature will be measured either by nasopharyngeal, rectal or urinary electronic temperature probe. The water heater will be adjusted to maintain the core temperature at 34°C. Once the cooling period has passed the baby will be rewarmed by increasing the target temperature by no more than 0.5°C every hour.

Cerebral function monitoring, using the aEEG, will be used from the onset of ECMO to both aid clinical management and provide additional information when assessing the outcome data.

During cooling heparin management will continue according to institutional protocols, however, it is important to note that the only study to use cooling for babies on ECMO was done using the Hemochron System (ITC, NJ, USA) and P214 tubes. The hemochron machine itself measures the activated clotting timeat 37°C. In addition none of the babies in this study received amicar, which should therefore not be administered during the NEST study. If an anti-fibrinolytic drug is indicated it is recommended that aprotinin is used with a loading dose of 1 ml/kg followed by an infusion of 1 ml/kg/hr.

### Outcomes

#### Primary outcome

Cognitive score from the Bayley scales of Infant and Toddler Development, 3rd edition (Bayley-III) [33] at age of 2 years (24 - 27 months).

#### Secondary outcomes

• Death

• Neurological optimality score [34,35]

• Gross and fine motor score from the Bayley-III [33]

• Cerebral Palsy

• Gross motor function classification score (GMFCS) [36]

• Seizures requiring regular anticonvulsant treatment

• Visual difficulties not corrected by spectacles

• Hearing difficulties requiring aids

• Language - expressive and receptive scores from the Bayley-III [33]

• Parent Report of Children's Abilities, (PARCA-R) [37-39]

• Infant Characteristics Questionnaire [40]

• The Brief Infant-Toddler Social and Emotional Assessment (BITSEA) [41]

• Measures of growth - height, weight and head circumference [10]

A child will be considered to be functioning within the normal range for age if their results are within the normal range for all Bayley scores and they have a normal neurological examination, normal vision (including with spectacles) and normal hearing (no aids).

### Neurodevelopmental tools and age at assessment

• The Bayley Scales at 2 years corrected age [33]. The Bayley Scales of Infant and Toddler Development are widely used for assessing development in young children. The scales have been recently revised and re-standardised and the administered scales are now in separated into three domains: cognition, language and motor (Bayley-III). For all three domains a score of 100 is average and scores of 115 and 85 represent respectively one standard deviation above and below the mean.

• The cognitive scale of the Bayley-III which has replaced the Mental Development Index (MDI) from the BSID-II, has the advantage of being relatively language free and will be used as the primary outcome measure. Language will be assessed using the language scale from the Bayley-III and also from a parent report (PARCA-R) [37-39]. The scales have good psychometric properties and reliability in the clinical setting. In a group of 850 three-year old term children in the UK, the BSID-II mean MDI was within 1 point of the US standardisation mean suggesting high relevance to the UK population [42]. The Bayley-III is currently being tested to check for UK norms and being using in several research contexts in the UK. Current outcome data from ECMO children finds few with very low cognitive levels and hence it should be possible to give a score to most children. Randomised trials of mild cooling as a neuroprotection measure following perinatal hypoxic-ischaemia have employed the Bayley score at around 2 years as a primary outcome [24]. Additionally the Bayley scales have been used for the follow up of preterm infants (EPICure [42] and PROGRAMS) at 2 years corrected age. Thus there is increasingly wide experience of using the Bayley scales in the UK. Cognitive abilities will also be assessed using the PARCA-R as a parent report measure. The PARCA was developed by Saudino *et al *[37] for use in term-born infants at two years, with additional items (PARCA-R) to assess non-verbal cognitive skills at a lower developmental level (validated by Johnson *et al *in preterms [38,39]).

• A formal clinical and neurological examination at 2 years corrected age [34,35]. This neurological exam gives an "optimality score" with a maximum score 78 (3 × 26 different items). The items are grouped into those relating to cranial nerve function (five), posture (six), tone (eight), movements (two) and reflexes (five). This score has been standardised to 18 months [34] but can be used in two year olds [43]. A functional description of the child's motor abilities will be recorded and whether they are thought to have a diagnosis of cerebral palsy. Motor function will also be assessed in term of gross and fine motor skills using the motor scale from the Bayley-III [33] and classified using the Gross Motor Function Classification score [36].

• Head circumference will also be measured. Centiles will be calculated related to neonatal head circumference centile [10]. Height and weight will also be measured.

• The administered Bayley scales will not give information about all aspects of development that are of concern in this group of infants. The existing data on outcome from ECMO eligible infants showed that 71% of survivors in both ECMO and control groups have no signs of impairment at one year of age; by age four years this proportion had fallen to 20% and 11.4% respectively [2] with overall cognitive function in the low normal range and specific difficulties with visuospatial tasks, behaviour and sensory modalities. Specific items in the Bayley-III scales assessing fine motor skills and visuospatial function will be looked at in detail. Given testing-time constraints it was felt that as the Bayley-III scales test these domains in more depth than the BSID-II we would not add further visuospatial testing as we originally suggested.

• Issues of behavioural problems (highlighted in the 4 year outcome study of the original ECMO trial cohort) will be assessed using Characteristics Questionnaire (ICQ) [40] and the BITSEA [41], parental report questionnaires. The ICQ assesses infant temperament and the BITSEA social-emotional and behavioural problems.

Data on short-term outcomes (up to discharge from hospital) will be collected during the babies' hospital stay. Major morbidity at 2 years corrected age will be assessed by a paediatrician, masked to study allocation.

### Long-term follow-up at 5 years

However, it is recognised that the detection of many subtle deficits, particularly in the fields of behaviour, attention and cognition, may be unreliable at the age of two years. These deficits have major implications for educational achievement and social functioning. We therefore hope to be able to perform a detailed, standardised clinical examination and assessment of neurological, developmental, cognitive and behavioural function at the age of five years. Families will be informed during recruitment of our intention to follow up their children again.

### Sample Size

Sample size estimates have been made based on what are considered to be potentially clinically important differences in the cognitive scores from the Bayley-III at two years corrected age.

Table 1 gives a range of total sample sizes based on variations in the mean and standard deviation of these scores.

**Table 1:** Sample size estimates

Assumed mean scores of the two arms	Assumed SD of Bayley-III cognitive scores	Total sample size required for 90% power	Number needed to be recruited assuming 80% survival to 2 years
85 & 95	15	94	118

85 & 95	10	42	53

90 & 95	10	168	210

The first of these options (requiring the recruitment of 118 infants) offers a realistic recruitment target whilst also giving 90% power to detect a significant difference between the two arms (at a two-tailed 5% level of significance). The choice of 85 and 95 as the two Bayley scores on which to derive the trial size is based on: a) what might be considered a clinically significant difference and b) existing knowledge of ECMO survivors [2,9].

### Feasibility

We estimate that it should be feasible to recruit this number of infants in 30 months as:

• all 4 UK ECMO centres have agreed to join the study.

• It is estimated that 70 suitable neonates will be referred to these centres per year (67% recruitment to the study is therefore required).

### Statistical Analysis

Demographic factors and clinical characteristics will be summarised with counts (percentages) for categorical variables, mean (standard deviation [SD]) for normally distributed continuous variables, or median (interquartile [IQR] or entire range) for other continuous variables.

Comparative statistical analysis will entail calculating the mean difference plus 95% confidence interval [CI] for the primary outcome (likewise for other normally distributed continuous outcomes), the median difference (plus 95% CI) for skewed continuous variables and the risk ratio (plus 95% CI) for secondary outcomes which are categorical.

For the primary analysis, patients will be analysed in the groups to which they are assigned, comparing the outcome of all babies allocated to "ECMO with cooling" with all those allocated to "ECMO" alone, regardless of deviation from the protocol or treatment received. For the primary outcome (the cognitive score from the Bayley-III at age 2 years), the analysis will compare the mean scores for each group of surviving babies. The rationale for this choice of primary analysis is to give a fair representation of the average ability of assessable children, accepting the limitation that excluding deaths might impose. There is potential for bias, but only in the presence of high and/or differential mortality between the groups. An adjusted analysis will be performed for the primary outcome to investigate the impact of stratification/known prognostic factors.

The primary analysis is therefore of survivors. Sensitivity analyses will be used to assess the robustness of the results and the impact of missing data. Missing primary outcomes data for surviving children arise due to (i) logistical reasons, (ii) parental wishes, (iii) children being untestable for behavioural reasons or (iv) children being too severely disabled to be assessed using the Bayley-III scales. Strategies employed will include:

1. imputing arbitrary scores

2. imputing scores using single imputation by using repeated random sampling from a distribution (mean = 100 SD = 15) to create a sample of scores per missing value e.g. 5, depending on the individual circumstances and impute the appropriate summary statistic i.e. the median.

For any children that we cannot see:

• but where we have some indication from parental questionnaire of no neurodevelopmental problems, we will sample randomly from those scores of children who score above -2 SDs of the mean

• but where we have no information regarding the child or there is an indication of a neurodevelopmental problem, then we would sample randomly from the entire range of scores within our study population

For any children we do see:

• but for whom we are unable to obtain a cognitive score on the Bayley-III because of behavioural difficulties, but who are motorically competent, we will sample from between 2 and 3 SDs below the mean

• but are too severely disabled to test, we will sample from between 3 and 4 SDs below the mean

NB. imputing missing values using multiple imputation techniques based on values of the population distribution (taking into account the child's most important characteristics) would be preferable, but the sample size in this study is too small.

A further sensitivity analysis will use rank based statistical methods; typically more conservative, but valid given that we are at the extremes of the distribution.

The consistency of the effect of cooling on the group of babies recruited to the trial will be explored to see whether cooling is of particular help, or not, to specific subgroups of infants, using the statistical test of interaction. Therefore pre-specified subgroup analyses include: (i) whether the ECMO is veno-arterial or veno-venous; (ii) whether the child's oxygenation index at the time of recruitment is <60 or ≥ 60; (iii) initial aEEG pattern shown on the cerebral function monitor, and (iv) primary diagnostic group. Subgroup analysis will be performed on the following outcomes (a) the primary outcome, (b) vision, (c) hearing and (d) neurological status. The subgroup analyses will be interpreted as exploratory.

### Ethics Approval

Ethics approval was granted by the Trent Research Ethics Committee of the UK National Research Ethics Service (reference: 05/MRE04/22).

### Organisation

#### Project management group

A core team of individuals will liaise regularly regarding the general progress and management of the study. This will normally occur by teleconference every 6 weeks. Trial Steering Committee meetings will occur annually. A central co-ordinating centre based at the National Perinatal Epidemiology Unit will be responsible for organising these meetings as well as:

• General oversight and compliance with procedures;

• Data management;

• Data analysis;

• Servicing the Data Monitoring Committee;

• Organising follow up appointments and maintaining contacts with families via newsletters and birthday cards.

#### Trial Steering Committee

The Trial Steering Committee will provide overall supervision of the trial on behalf of the British Heart Foundation. Its terms of reference are:

• To monitor and supervise the progress of the NEST study towards its interim and overall objectives;

• To review at regular intervals relevant information from other sources (related studies);

• To consider the recommendations of the Data Monitoring Committee;

• In the light of 1, 2 and 3 above to inform the British Heart Foundation of progress of the study;

• To advise the British Heart Foundation on publicity and the presentation of all aspects of the study.

The membership is:

Professor Andrew Wilkinson (Chair), Consultant Paediatrician, John Radcliffe Hospital, Oxford.

Professor Marianne Thoresen, Professor of Neonatal Neuroscience, University of Bristol.

Dr Clare Snowdon, Research Fellow, LSHTM, London

Ms Pauline Fellows, NSC Neonatal Project Facilitator, Addenbrooke's Hospital, Cambridge

Ms Farrah Pradhan, Family Support Co-ordinator, Bliss, London

Dr Mary Montgomery, Consultant in Retrieval and Critical Care, Children's Acute Transport Services and the Royal Brompton Hospital PICU, London

Professor David Field (Chief Investigator), Consultant Paediatrician, Dept of Health Sciences, University of Leicester

Professor Peter Brocklehurst (Investigator), Director, NPEU, Oxford

#### Data Monitoring Committee

This will be independent of the study organisers and will meet annually. During the period of recruitment to the study, interim analyses will be supplied, in strict confidence, to the DMC, together with any other analyses the DMC may request. The data will be supplied to the Chair of the DMC as frequently as she requests. Meetings of the committee will be arranged periodically, as considered appropriate by the Chair. In the light of interim data, and other evidence from relevant studies (including updated overviews of the relevant randomised controlled trials), the DMC will inform the Steering Committee, if in their view i) there is proof beyond reasonable doubt that the data indicate that any part of the protocol under investigation is either clearly indicated or contra-indicated, either for all infants or for a particular subgroup of trial participants or ii) it is evident that no clear outcome will be obtained.

Appropriate criteria for proof beyond reasonable doubt cannot be specified precisely. A difference of at least 3 standard errors in the interim analysis of a major endpoint may be needed to justify halting, or modifying, such a study prematurely. If this criterion were to be adopted, it would have the practical advantage that the exact number of interim analyses would be of little importance. One interim analysis is planned for each year of recruitment, as well as one analysis six months after recruitment has stopped. These analyses will concentrate on outcomes at hospital discharge, as many of these babies will not have had their two-year assessments performed at the time of the interim analyses. Unless modification or cessation of the protocol is recommended by the DMC, the Trial Steering Committee, collaborators and administrative staff (except those who supply the confidential information) will remain ignorant of the results of the interim analysis. Collaborators and all others associated with the study may write through the NEST Co-ordinating Centre to the DMC to draw attention to any concern they may have about the possibility of harm arising from the treatment under study, or about any other matters that may be relevant.

The membership is:

Professor Diana Elbourne (Chair), Prof. Health Care Evaluation, LSHTM, London

Dr Duncan Macrae, Director of PIC, Royal Brompton Hospital, London

Professor Henry Halliday, Consultant Paediatrician, Royal Maternity Hospital, Belfast

Professor Richard Cooke, Consultant Paediatrician, Liverpool Women's Hospital.

Professor Neil Marlow, Professor of Neonatal Medicine, UCL Institute For Women's Health, London

Professor Linda Franck, Prof. and Chair Children's Nursing Research Centre for Nursing and Allied Health Professions Research, Great Ormond Street Hospital, London

Serious Adverse Events and Suspected Unexpected Serious Adverse Reaction reporting

#### Assessment of Safety

Safety monitoring has been delegated by the Sponsor (University Hospitals of Leicester NHS Trust, of Leicester) to the National Perinatal Epidemiology Unit (NPEU) at the University of Oxford.

Safety will be assessed continuously during each baby's stay in the ECMO unit. Any adverse events which require expedited reporting will follow the system outlined below.

Other outcomes, which may also be considered safety outcomes, such as early neonatal morbidity, but which are anticipated outcomes for this group of babies, will be captured on study specific data collection forms.

#### Expected Adverse Drug Reactions

No adverse drug reactions will occur as the study intervention is not a drug.

#### Expected Serious Adverse Events (SAEs)

The following are serious adverse events that could be reasonably expected for this group of babies during the course of the study:

• Death

• Major ECMO circuit complications such as oxygenation failure of line rupture

• Major cerebral haemorrhages after trial entry

• Major haemorrhage requiring transfusion

For the purposes of this study these SAEs require immediate reporting.

#### Serious Adverse Reactions (SARs)

Although the study intervention is not a drug, the only theoretically possible recognised adverse reactions/events associated with this treatment are:

• Deranged clotting arising during cooling period

• Evidence of impaired immunity arising during cooling period

#### Causality Assessment

All cases judged by either the reporting medically qualified person or the Chief Investigator as having a reasonable suspected causal relationship to the treatment qualify as an Adverse Event.

#### Expedited/immediate reporting of safety events

All expected SAEs or SARs described above must be reported to the Trial Co-ordinating Centre within 10 days of discovery or notification of the event, this will be immediately referred to the Chief Investigator or his delegated deputy. If urgent safety measures will be required as a result of an SAE or SAR then the event or reaction must be reported within one working day of discovery or notification of the event to the Trial Co-ordinating Centre. A Standard Operating Procedure (SOP) outlining the reporting procedure for clinicians will be provided on the reverse of the SAE form. An SOP will also be available as part of the Trial Specific SOPs which will outline the reporting procedure for the Trial Co-ordinating Centre. All SAE and SAR information must be recorded on an SAE form and faxed to the Chief Investigator or their delegated Deputy. Additional information received for a case (follow-up or corrections to the original case) needs to be detailed on a new SAE and SAR form and faxed to the Chief Investigator or their delegated deputy.

The severity of events will be assessed on the following scale: 1 = mild, 2 = moderate, 3 = severe. The relationship of SAEs or SARs to the study intervention will be assessed according to the definition provided above.

The Trial Co-ordinating Centre will report all SAEs and SARs to the Ethics Committee concerned using the SAE report form for non-CTIMPs, available from the NRES website. In addition a copy of the SAE or SAR will be forwarded to the Chair of the Data Monitoring Committee. The Chair will also be provided with a document detailing all previous SAEs and SARs. The Chief Investigator will also inform all investigators concerned of relevant information about SAEs and SARs that could adversely affect the safety of participants.

In addition to the expedited reporting above, the Chief Investigator shall submit, once a year throughout the clinical trial, or on request, a safety report to the Ethics Committee.

#### Reporting procedures for all adverse events

All adverse events (AEs) occurring during the study observed by the investigator or reported by the participant, whether or not attributed to study intervention, will be reported on the data collection form. AEs considered to be related to the study intervention by the investigator will be followed up until resolution or the event is considered stable. The investigator may be asked to provide follow-up information.

All related AEs that result in a participant's withdrawal from the study or are present at the end of the study, should be followed up until a satisfactory resolution occurs.

It will be left to the investigator's clinical judgment whether or not an AE is of sufficient severity to require the participant's removal from treatment. A participant may also be voluntarily withdrawn from treatment due to what the attending clinician or the parents perceive to be an intolerable AE.

### Publication policy

The Chief Investigator will co-ordinate dissemination of data from this study. All publications using data from this study to undertake original analyses will be submitted to the Trial Steering Committee (TSC) for review before release.

To safeguard the scientific integrity of the trial, data from this study will not be presented in public before the main results are published without the prior consent of the TSC. The success of the trial depends on a number of neonatal ECMO specialists, intensivists and parents. For this reason, chief credit for the results will not be given to the committees or central organisers, but to all who have collaborated and participated in the study. Acknowledgement will include all local co-ordinators and collaborators, members of the trial committees, the Trial Co-ordinating Centre and trial staff. Authorship at the head of the primary results paper will take the form "The NEST Study Collaborative Group". This avoids giving undue prominence to any individual. All contributors to the study will be listed at the end of the report, with their contribution to the study identified.

Those responsible for other publications reporting specific aspects of the study may wish to utilise a different authorship model, such as " [name], [name] and [name] on behalf of the NEST Study Collaborative Group". Decisions about authorship of additional papers will be discussed and agreed by the trial investigators and the TSC.

Parents will be sent a summary of the final results of the study unless they have indicated previously that they do not wish to receive such a summary. Opportunities to opt out from receiving information about the progress of the study and/or a summary of the results will be offered periodically with the parents' newsletters. The summary will contain a reference to the full paper. A copy of the journal article will be available on request from the NPEU.

## Competing interests

The authors declare that they have no competing interests.

## Authors' contributions

DF lead the overall project, PB and EJ advised on study design and provided statistical expertise, DA advised on the clinical aspects of cooling, FC lead the development of the approach to follow up and RF provided clinical expertise in relation to ECMO. All authors contributed to the drafting of the manuscript and have approved the final version.

## Pre-publication history

The pre-publication history for this paper can be accessed here:

http://www.biomedcentral.com/1471-2431/10/24/prepub
